# FAN score comprising fibrosis-4 index, albumin–bilirubin score and neutrophil–lymphocyte ratio is a prognostic marker of urothelial carcinoma patients treated with pembrolizumab

**DOI:** 10.1038/s41598-021-00509-x

**Published:** 2021-10-27

**Authors:** Atsunari Kawashima, Yoshiyuki Yamamoto, Mototaka Sato, Wataru Nakata, Yoichi Kakuta, Yu Ishizuya, Yuichiro Yamaguchi, Akinaru Yamamoto, Takahiro Yoshida, Hitoshi Takayama, Tsuyoshi Takada, Hitoshi Inoue, Yohei Okuda, Taigo Kato, Koji Hatano, Motohide Uemura, Norio Nonomura, Ryoichi Imamura

**Affiliations:** 1 Department of Urology, Osaka University Graduate School of Medicine, 2-2 Yamadaoka, Suita, Osaka 565-0871 Japan; 2 Department of Urology, Osaka International Cancer Institute, Osaka, Japan; 3 Department of Urology, Toyonaka Municipal Hospital, Toyonaka, Japan; 4 Department of Urology, Osaka Rosai Hospital, Sakai, Japan; 5 Department of Urology, Osaka General Medical Center, Osaka, Japan; 6 Department of Urology, Higashiosaka City Medical Center, Higashiōsaka, Japan; 7 Department of Urology, JCHO Osaka Hospital, Osaka, Japan; 8 Department of Urology, Osaka Police Hospital, Osaka, Japan; 9 Department of Urology, Hyogo Prefectural Nishinomiya Hospital, Nishinomiya, Japan; 10 Department of Urology, Sakai City Medical Center, Sakai, Japan; 11 Department of Urology, Minoh City Hospital, Minoh, Japan; 12 Department of Urology, Ikeda City Hospital, Ikeda, Japan; 13 Department of Urology, Sumitomo Hospital, Osaka, Japan

**Keywords:** Tumour immunology, Urological cancer, Cancer

## Abstract

It is important to identify prognostic and predictive markers of metastatic urothelial carcinoma (mUC) treated with immunocheckpoint inhibitors. We sought to establish a prognostic marker for patients with mUC treated with pembrolizumab based on only blood test results. We included 165 patients with mUC in the discovery cohort and 103 with mUC who were treated with pembrolizumab in the validation cohort. Multivariate and Cox regression analyses were used to analyse the data. In the discovery cohort, the fibrosis-4 index (hazard ratio [HR]: 2.13, 95% confidence interval [CI] 1.20–3.76, p = 0.010), albumin–bilirubin score (HR 1.91, 95% CI 1.27–2.88, p = 0.002), and neutrophil–lymphocyte ratio (HR: 1.84, 95% CI 1.22–2.79, p = 0.004) were independent significant prognostic factors. We established a ‘FAN score’ that included these three aforementioned items, which were assigned one point each. We divided patients into the 0–1 point (n = 116) and 2–3 points (n = 49) groups. The FAN score was a significant prognostic marker for cancer-specific survival (CSS) (HR 1.48, 95% CI 1.19–1.83, p < 0.001) along with the Eastern Cooperative Oncology Group Performance Status. The FAN score was also a prognostic factor of progression-free survival (PFS) (HR: 1.25, 95% CI 1.01–1.54, p = 0.036) along with the presence of liver metastasis. In the validation cohort, the FAN score was a significant prognostic factor for CSS (HR: 1.48, 95% CI 1.19–1.85, p = 0.001) and PFS (HR: 1.29, 95% CI 1.02–1.62, p = 0.034). We established the FAN score as a prognostic marker for patients with mUC treated with pembrolizumab.

## Introduction

Immunotherapy targeting immunocheckpoint molecules (ICI therapy) has been clinically used as a mainly second-line treatment after platinum-based chemotherapy for patients with metastatic and advanced urothelial carcinoma (UC) to improve prognosis^1–3^. However, its therapeutic efficacy is limited and severe immune-related adverse events occur in some patients. Thus, it is important to establish a useful and simple biomarker that can easily predict the efficacy and prognosis of ICI therapy to obtain a more effective therapeutic effect^4–7^.

Several prognostic models have been reported for ICI therapy for metastatic UC (mUC) including the neutrophil-to-lymphocyte ratio (NLR)^8–11^, anaemia^10^, metastatic sites^8–12^, and/or performance status^8–12^. However, no prognostic models that use only blood test results have been created.

Recently, several studies have reported attempts to establish a diagnostic and prognostic model based on blood test results in daily clinical practice using machine learning techniques with the artificial intelligence^13^. However, the rationale is usually unknown because of the black box problem. Therefore, it is still necessary to develop a clear clinical model based on evaluations of biological function by human intelligence.

It well known the presence of liver metastasis to become a poor prognostic factor of mUC patients treated with chemotherapy and ICI therapy commonly. The aforementioned poor prognostic factors of mUC patients are associated with cancer cachexia related to liver dysfunction. So, we supposed that scoring system reflecting liver function could be useful for establishing a prognostic model using blood test.

So, we sought to establish a prognostic marker for patients with mUC treated with pembrolizumab based on only blood test results, including the scoring system reflecting liver function in this study.

## Materials and methods

### Study design and population

In this study, we aimed to establish a significant prognostic model using only blood tests through two independent cohorts. First, we collected and analysed blood test results of 165 patients with mUC from six institutions as a discovery cohort. After establishing a significant prognostic model in multivariate analysis, we blindly collected clinical data and evaluated its validity in 103 patients with mUC from seven institutions as a validation cohort (Supplemental Fig. 1).

Totally, we retrospectively analysed a database comprising 268 patients treated from April 2016 to December 2020 with pembrolizumab as second-line or later treatment for mUC at Osaka University Graduate School of Medicine and its affiliated hospitals listed in the acknowledgements.

This study was approved by the institutional review board of Osaka University, which provided the necessary institutional data-sharing agreements before initiation of the study (#19083), and the study was conducted in accordance with the Declaration of Helsinki.

### Data collection

The patient characteristics including laboratory findings were evaluated at the time of drug administration. Clinical features evaluated were age, sex, the Eastern Cooperative Oncology Group Performance Status (ECOG PS), levels of haemoglobin, serum albumin, serum bilirubin, serum aspartate aminotransferase (AST), serum alanine aminotransferase (ALT), estimated glomerular filtration rate (eGFR)^14^, and C-reactive protein (CRP), the presence of radical surgery, sites and number of organs involved in metastasis, and counts of leukocytes, monocytes, and platelets. The fibrosis-4 (Fib-4) index is reported as a non-invasive liver fibrosis marker and was calculated using the formula: age (years) × AST (U/L)/(platelet count [103/ μL] × (ALT [U/L]0.5)^15^. The albumin–bilirubin (ALBI) score has been proposed as a validated index of liver dysfunction and was calculated using the formula: log10 (T-bil [mg/dL] × 17.1) × 0.66 + albumin [mg/dL] × 10(− 0.085)^16^. The neutrophil–lymphocyte ratio (NLR) was calculated using the formula: neutrophil count/ lymphocyte count. The following cut-off values were defined according to previous reports: Fib-4 > 3.5^17^, ALBI score > − 2.6^16^, and NLR > 5.0^8^.

The proximal efficacy of pembrolizumab was evaluated based on RECIST 1.1^18^. Cancer-specific survival (CSS) time was calculated from the date of initiation of pembrolizumab until death or the date of the patient’s last follow-up visit. Progression-free survival (PFS) was calculated from the date of initiation of pembrolizumab until radiological and clinical disease progression or death. The CSS and PFS rates were calculated using the Kaplan–Meier method.

### Establishment of the prognostic prediction model based on the blood test results

The evaluated items in this analysis were haemoglobin level (< 10 g/dL or ≥ 10 g/dL), platelet count (> 32.0 × 10^4^ × /μL or ≤ 32.0 × 10^4^ × /μL), AST level (< 40 U/L or ≥ 40 U/L), ALT level (< 40 U/L or ≥ 40 U/L), serum sodium level (≥ 138 mEq/L or ≤ 137 mEq/L), eGFR (< 60 mL/min/1.73 m^2^ or ≥ 60 mL/min/1.73 m^2^), albumin level (≥ 3.5 mg/dL or < 3.5 mg/dL), CRP level (< 1.0 mg/dL or ≥ 1.0 mg/dL), monocyte-lymphocyte ratio (≤ median or > median), platelet-lymphocyte ratio (≤ median or > median), NLR (≥ 5.0 or < 5.0), Fib-4 index (≥ 3.5 or < 3.5), and ALBI score (> − 2.6 or ≤ − 2.6) (Supplemental Table 1).

To establish the prognostic prediction model for CSS, only blood test results were evaluated using Cox regression analysis with stepwise regression backward and forward selection with p < 0.05 as the criterion for model entry or continued inclusion in multivariate analysis.

### Statistical analysis

Clinical items in the multivariate analysis were sex (male or female), the primary site (upper urinary tract involvement or others), surgical removal of the primary site (yes or no), previous chemotherapy regimen (cisplatin based or non-cisplatin based), time from the last chemotherapy (< 90 days or ≥ 90 days), ECOG PS (0, 1, or ≥ 2), organs involved in metastasis (only lymph nodes, liver metastasis, or other organs), discontinuation of pembrolizumab due to adverse events (yes or no), presence of anaemia, and presence of renal dysfunction. We used the Cox regression model to calculate the hazard ratios (HRs) in univariate and multivariate analyses. Prognostic factors related to CSS and PFS were analysed using Cox regression analysis, and statistical significance was set as p < 0.05. Statistical analyses were performed using the Statistical Package for the Social Sciences software, version 20.0 (IBM Corp., Armonk, NY, USA).

### Ethics approval

This study was approved by the institutional review board of Osaka University, which provided the necessary institutional data-sharing agreements before initiation of the study (#19083), and the study was conducted in accordance with the Declaration of Helsinki.

### Consent to participate

Informed consent was obtained from all individual participants included in the study.

## Results

### Patient characteristics

The clinical characteristics of the 268 patients with mUC enrolled in this study are shown in Table 1. Median patient age was 73 (range, 28–93) years, and primary cancer sites were the bladder (106 patients), upper urinary tract (157 patients), and both (5 patients). The numbers of patients with a high Fib-4 index, ALBI score, and NLR were 22 (8.2%), 134 (50%), and 83 (31%), respectively. Only the rate of surgical removal at the primary site was significantly different between the two cohorts (p < 0.001). The median duration of treatment with pembrolizumab was 3.22 (0.26–35.9 months), and the median follow-up period was 6.69 (0.26–37.0) months.

**Table 1:** Clinical characteristics of 268 patients in this study.

Characteristic	Total	Discovery cohort	Validation cohort	p
	*n* = 268	*n* = 165	*n* = 103	
Age, years, range (median)	28–93 (73)	28–93 (73)	30–86 (73)	0.418
**Sex, N (%)**				
Male	193 (72.0)	117 (70.9)	76 (73.8)	0.676
Female	75 (28.0)	48 (29.1)	27 (26.2)	
**Primary site, N (%)**				
Bladder	106 (39.6)	61 (37.0)	45 (43.7)	0.136
Upper urinary tract	157 (58.9)	99 (60.0)	58 (56.3)	
Both	5 (1.5)	5 (3.0)	0 (0)	
**Surgical removal of primary site, N (%)**				
Yes	181 (67.5)	98 (59.4)	83 (80.6)	< 0.001
No	87 (32.5)	67 (40.6)	20 (19.4)	
**Previous chemotherapy regimen, N (%)**				
Cisplatin based	160 (59.7)	105 (63.6)	55 (53.4)	0.124
Non cisplatin based	108 (40.3)	60 (36.4)	48 (46.6)	
**Time from last chemotherapy, N (%)**				
< 90 days	164 (61.2)	96 (58.2)	68 (66.0)	0.248
≥ 90 days	104 (38.8)	69 (41.8)	35 (34.0)	
**ECOG performance status, N (%)**				
0	80 (29.9)	58 (35.2)	22 (21.4)	0.050
1	138 (51.5)	80 (48.5)	58 (56.3)	
≥ 2	50 (18.6)	27 (16.3)	23 (22.3)	
**Metastatic sites, N (%)**				
Lymphnodes only	65 (24.3)	43 (26.1)	22 (21.4)	0.673
Other organs	144 (53.7)	86 (52.1)	58 (56.3)	
Liver metastasis	59 (22.0)	36 (21.8)	23 (22.3)	
**eGFR (ml/min/1.73 m**^**2**^**), N (%)**				
≥ 60.0	54 (20.1)	31 (18.8)	23 (22.3)	0.922
30.0 ≤ , < 60.0	173 (64.6)	109 (66.1)	64 (62.1)	
< 30.0	41 (15.3)	25 (15.1)	16 (15.6)	
**Hb (g/dL), N (%)**				
< 10.0	134 (50.0)	97 (58.8)	37 (35.9)	0.388
≥ 10.0	134 (50.0)	68 (41.2)	66 (64.1)	
**Fib-4 index, N (%)**				
< 3.5	246 (91.8)	150 (90.9)	96 (93.2)	0.649
≥ 3.5	22 (8.2)	15 (9.1)	7 (6.8)	
**ALBI score, N (%)**				
≤ − 2.6	134 (50.0)	76 (46.1)	58 (56.3)	0.801
> − 2.6	134 (50.0)	89 (53.9)	45 (43.7)	
**NLR, N (%)**				
< 5.0	185 (69.0)	111 (67.3)	74 (71.8)	0.498
≥ 5.0	83 (31.0)	54 (32.7)	29 (28.2)	
**FAN score, N (%)**				
Low	188 (70.1)	115 (69.7)	73 (70.9)	0.891
High	80 (29.9)	50 (30.3)	30 (29.1)	
**Discontinuation due to AEs, N (%)**				
No	216 (80.6)	138 (83.6)	78 (75.7)	0.515
Yes	52 (19.4)	27 (16.4)	25 (24.3)	
Treatment period, months, range (median)	0.26–35.9 (3.22)	0.26–35.9 (3.01)	0.99–34.1 (3.47)	0.977
Follow-up period, months, range (median)	0.26–37.0 (6.69)	0.26–37.0 (6.71)	0.99–36.1 (6.67)	0.821

ECOG: Eastern Cooperative Oncology Group, eGFR: estimated glomerular filtration rate, Fib-4 index: fibrosis-4 index, ALBI score: albumin–bilirubin score, NLR: neutrophil–lymphocyte ratio, FAN: Fib-4 index, ALBI score and NLR, AEs: adverse events.

### Treatment results in the two independent cohorts

**Figure 1:**
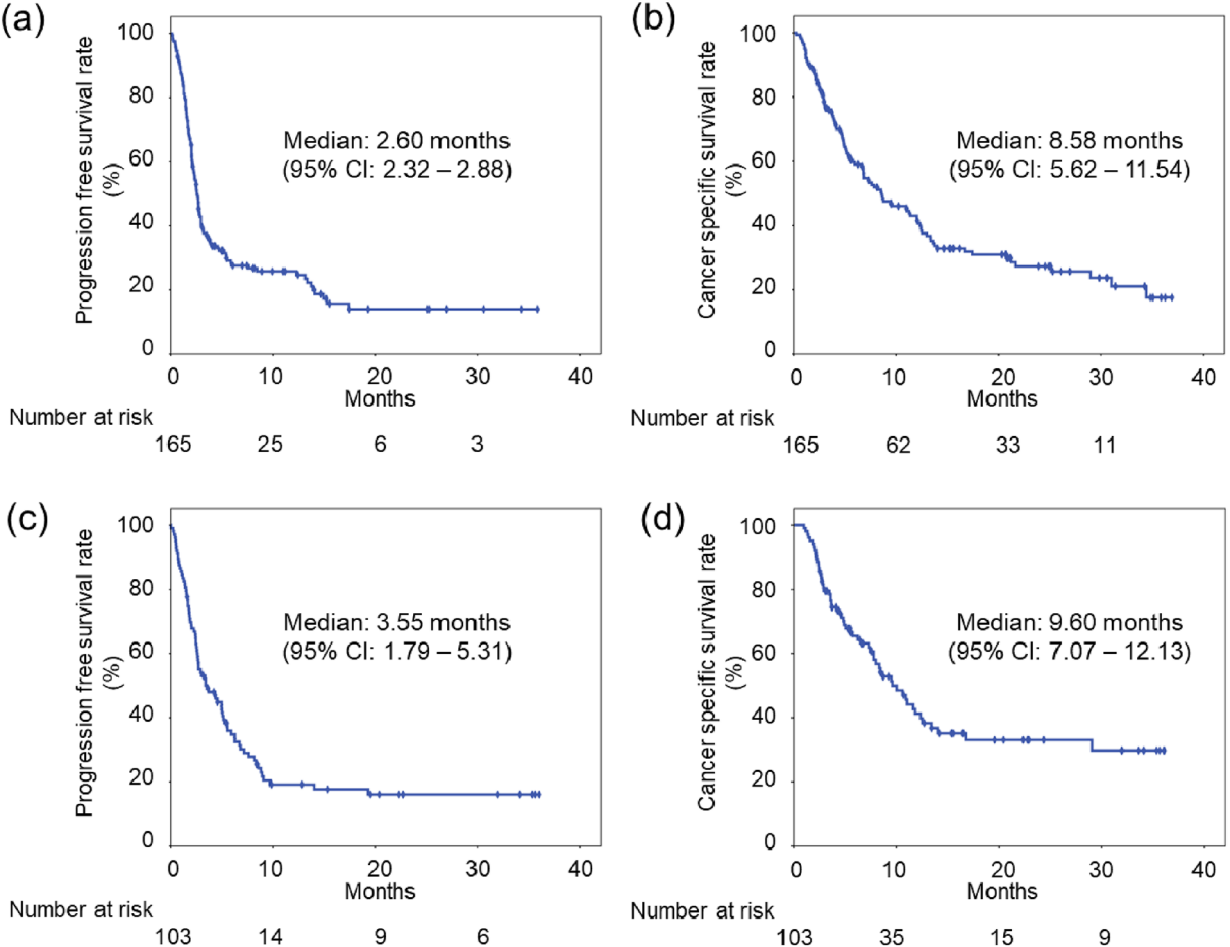
Probability estimates of the prognosis of the study patients with metastatic urothelial carcinoma who were treated with pembrolizumab. Probability estimates of progression-free survival (PFS) (**a**) and cancer-specific survival (CSS) (**b**) for the 165 patients in the discovery cohort. Probability estimates of PFS (**c**) and CSS (**d**) for the 103 patients in the validation cohort. CI, confidence interval.

In the discovery cohort, the median PFS and CSS were 2.60 months (95% confidence interval [CI] 2.32–2.88) and 8.58 months (95% CI 5.62–11.54) (Fig. 1a,b). Complete response (CR), partial response (PR), and stable disease (SD) were achieved in 14 patients (8.5%), 22 patients (13.3%), and 28 patients (17.0%), respectively. The objective response rate (ORR) was 21.8%, and the disease control rate (DCR) was 38.8% (Supplemental Table 2).

In the validation cohort, the median PFS and CSS were 3.55 months (95% CI 1.79–5.31) and 9.60 months (95% CI 7.07–12.13) (Fig. 1c,d). CR, PR, and SD were achieved in four patients (3.9%), 27 patients (26.2%), and 20 patients (19.4%), respectively. The ORR was 30.1%, and the DCR was 49.5% (Supplemental Table 2).

### Establishment of the FAN score

**Figure 2:**
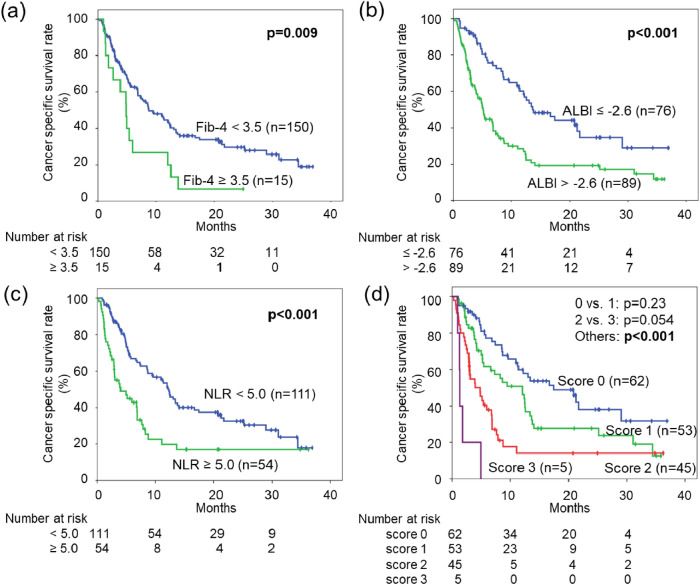
Probability estimates of the cancer-specific survival rate for the 165 patients in the discovery cohort stratified by three independent prognostic factors (fibrosis-4 [Fib-4] index (**a**), albumin–bilirubin [ALBI] score (**b**), and neutrophil-to-lymphocyte ratio [NLR] (**c**)) and the FAN score, which consisted of these three aforementioned prognostic factors.

Cox regression analysis identified three independent significant prognostic factors for CSS by stepwise backward analysis. The prognosis of patients with a high Fib-4 index (HR: 2.13, 95% CI 1.20–3.76, p = 0.010), high ALBI score (HR: 1.91, 95% CI 1.27–2.88, p = 0.002), and high NLR (HR: 1.84, 95% CI 1.22–2.79, p = 0.004) was significantly poor (Fig. 2a–c, Table 2). Because the HRs of these three prognostic factors were almost the same, we defined the FAN score (Fib-4 index, ALBI score, and NLR) as a prognostic factor by assigning one point to each item and compared the prognosis between the patients grouped by points. Although the prognosis became poorer as the score increased, there was no significant difference between scores of 0 and 1 and scores of 2 and 3 after multiple comparisons (Fig. 2d).

**Table 2:** Significant prognostic factors for cancer specific survival of metastatic urothelial carcinoma patients treated with pembrolizumab identified by Cox regression analysis with stepwise regression analysis in discovery cohort (n = 165).

	Univariate				Multivariate			
	HR	95% CI		p-value	HR	95%CI		p-value
		Lower	Higher			Lower	Higher	
Fib-4 index (≥ 3.5 vs. < 3.5)	2.08	1.18	3.66	0.011	2.13	1.20	3.76	0.010
ALBI score (> − 2.6 vs. ≤ − 2.6)	2.24	1.51	3.33	< 0.001	1.91	1.27	2.88	0.002
NLR (≥ 5.0 vs. < 5.0)	2.14	1.44	3.19	< 0.001	1.84	1.22	2.79	0.004

Fib-4 index: fibrosis-4 index, ALBI score: albumin–bilirubin score, NLR: neutrophil–lymphocyte ratio, HR: hazard ratio, CI: confidence interval.

**Figure 3:**
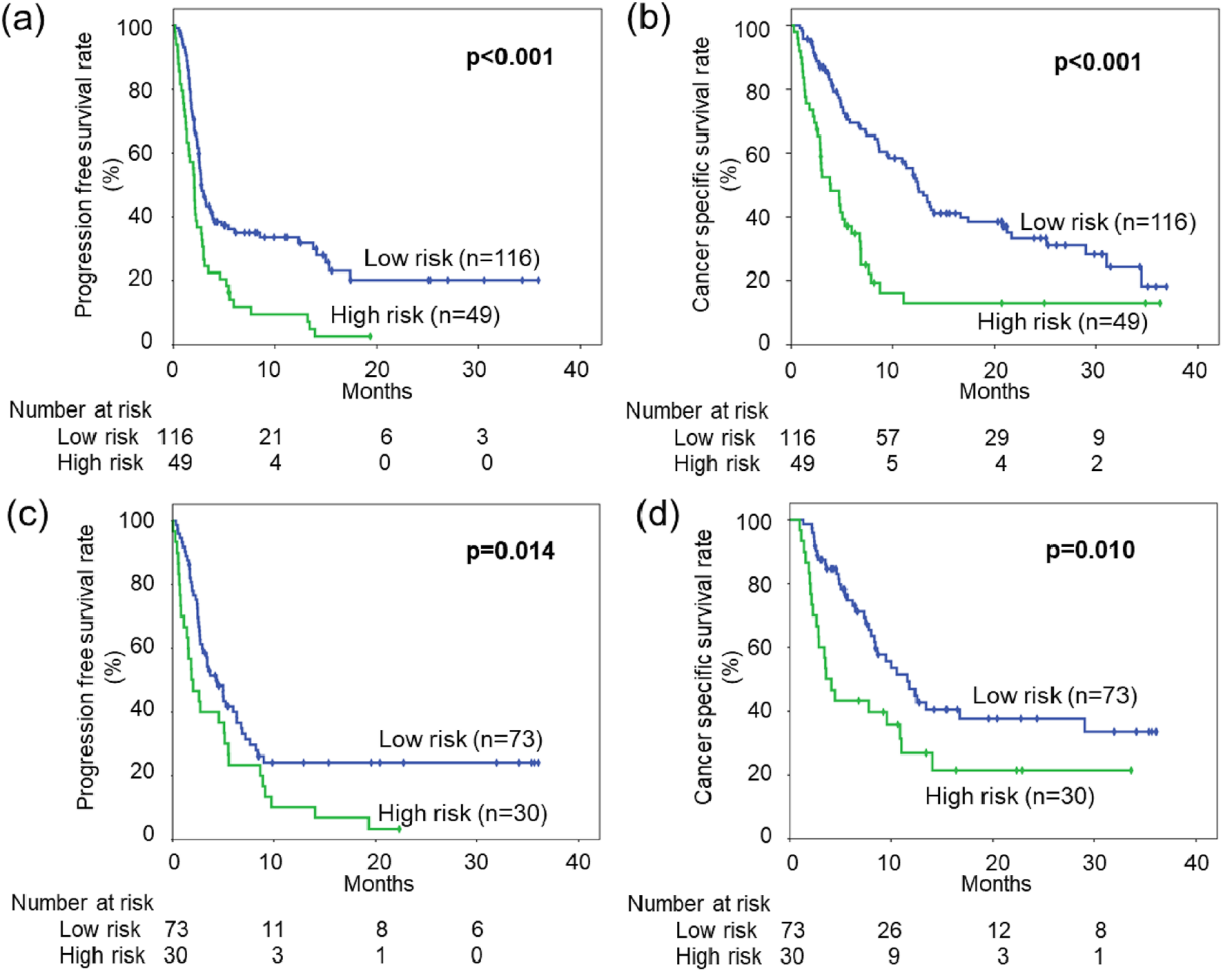
Probability estimates of the prognosis of study patients with metastatic urothelial carcinoma who were treated with pembrolizumab and stratified by the FAN score. Probability estimates of progression-free survival (PFS) (**a**) and cancer-specific survival (CSS) (**b**) for the 165 patients stratified by the FAN score in the discovery cohort. Probability estimates of PFS (**c**) and CSS (**d**) for the 103 patients stratified by the FAN score in the validation cohort.

Therefore, we classified scores of 0 and 1 as the low-risk group and scores of 2 and 3 as the high-risk group, and then evaluated its usefulness as a prognostic factor. The median PFS durations of the low-risk and high-risk groups were 2.76 months (95% CI 2.24–3.28) and 2.07 months (95% CI 1.89–2.25) (p < 0.001) (Fig. 3a). Moreover, the median CSS durations of the low-risk and high-risk groups were 12.4 months (95% CI 10.6–14.2) and 3.91 months (95% CI 1.9–5.91) (p < 0.001) (Fig. 3b).

### Clinical importance of the FAN score in the discovery cohort

About clinical therapeutic response, ORR was 25.9% and 12.2% in low-risk and high-risk group based on the FAN score, respectively. Also, DCR was 48.3% and 16.3% in low-risk and high-risk group based on the FAN score, respectively and DCR was significantly higher in low-risk group base on the FAN score (p < 0.001) (Supplemental Table 3).

Regarding PFS, short time from the last chemotherapy, anaemia, the presence of liver metastasis, and high-risk group based on the FAN score were associated with poor prognosis in univariate analysis (Table 3). In multivariate analysis, the presence of liver metastasis (HR: 1.93; 95% CI 1.13–3.23, p = 0.018) and high-risk group based on the FAN score (HR: 1.25, 95% CI 1.01–1.54, p = 0.036) were significant prognostic factors for predicting an inferior PFS (Table 3).

**Table 3:** Univariate and multivariate analysis of predictive factors for PFS in discovery cohort (n = 165).

	Univariate				Multivariate			
	HR	95% CI		p	HR	95%CI		p
		Lower	Higher			Lower	Higher	
**Age**								
< 73 vs. ≥ 73	1.05	0.74	1.49	0.786	0.96	0.67	1.38	0.814
**Sex**								
Male vs. female	1.39	0.95	2.01	0.088	1.21	0.82	1.79	0.330
**Primary site**								
Upper urinary tract involvement vs. others	1.02	0.71	1.47	0.899				
**Surgical removal of primary site**								
No vs. yes	0.75	0.53	1.07	0.113				
**Previous chemotherapy regimen**								
Cisplatin vs. non-cisplatin	0.83	0.58	1.20	0.327				
**Time from last chemotherapy (days)**								
≥ 90 vs. < 90	1.46	1.02	2.09	0.040	1.32	0.91	1.92	0.151
**Hb (g/dl)**								
≥ 10.0 vs. < 10.0	1.89	1.33	2.68	< 0.001	1.34	0.89	2.01	0.156
**ECOG performance status**								
0 vs. 1	1.21	0.82	1.80	0.344				
0 vs. ≥ 2	1.54	0.93	2.54	0.091				
**Metastatic sites**								
Lymphnode vs. other organs	1.15	0.75	1.77	0.524	1.18	0.76	1.83	0.469
Lymphnode vs. liver metastasis	2.28	1.37	3.82	0.002	1.93	1.13	3.23	0.018
**eGFR (ml/min/1.73 m**^**2**^**)**								
≥ 60 vs. 30.0 ≤ , < 60.0	0.70	0.45	1.10	0.124				
≥ 60 vs. < 30	0.88	0.50	1.56	0.665				
**FAN score**								
Low risk group vs. high risk group	1.43	1.19	1.72	< 0.001	1.25	1.01	1.54	0.036

Hb: hemoglobin, ECOG: Eastern Cooperative Oncology Group, eGFR: estimated glomerular filtration rate, FAN: Fib-4 index, ALBI score and NLR, HR: hazard ratio, CI: confidence interval.

Regarding CSS, short time from the last chemotherapy, anaemia, poor ECOG PS, the presence of liver metastasis, and high-risk group based on the FAN score were associated with poor prognosis in univariate analysis (Table 4). In multivariate analysis, poor ECOG PS (0 versus [vs.] 1, HR: 2.32; 95% CI 1.44–3.72, p = 0.001; 0 vs. 2, HR: 3.05; 95% CI 1.70–5.47, p < 0.001) and high-risk group based on the FAN score (HR: 1.48, 95% CI 1.19–1.83, p < 0.001) were significant prognostic factors for predicting an inferior CSS (Table 4).

**Table 4:** Univariate and multivariate analysis of predictive factors for CSS in discovery cohort (n = 165).

	Univariate				Multivariate			
	HR	95% CI		p	HR	95%CI		p
		Lower	Higher			Lower	Higher	
**Age**								
< 73 vs. ≥ 73	1.26	0.86	1.84	0.240	1.19	0.79	1.79	0.409
**Sex**								
Male vs. female	1.47	0.98	2.19	0.061	1.28	0.85	1.94	0.238
**Primary site**								
Upper urinary tract involvement vs. others	1.10	0.74	1.63	0.629				
**Surgical removal of primary site**								
no vs. yes	0.72	0.49	1.06	0.092				
**Previous chemotherapy regimen**								
Cisplatin vs. non-cisplatin	1.05	0.71	1.55	0.805				
**Time from last chemotherapy (days)**								
≥ 90 vs. < 90	1.57	1.06	2.32	0.025	1.29	0.85	1.96	0.227
**Hb (g/dl)**								
≥ 10.0 vs. < 10.0	1.73	1.18	2.53	< 0.001	1.26	0.83	1.91	0.287
**ECOG performance status**								
0 vs. 1	2.28	1.44	3.62	< 0.001	2.32	1.44	3.72	0.001
0 vs. ≥ 2	3.33	1.89	5.85	< 0.001	3.05	1.70	5.47	< 0.001
**Metastatic sites**								
Lymphnode vs. other organs	1.17	0.74	1.85	0.509	1.04	0.65	1.66	0.884
Lymphnode vs. liver metastasis	1.95	1.13	3.38	0.017	1.48	0.84	2.62	0.180
**eGFR (ml/min/1.73 m**^**2**^**)**								
≥ 60 vs. 30.0 ≤ , < 60.0	0.78	0.48	1.29	0.665				
≥ 60 vs. < 30	1.41	0.75	2.62	0.283				
**FAN score**								
Low risk group vs. high risk group	1.61	1.32	1.98	< 0.001	1.48	1.19	1.83	< 0.001

Hb: hemoglobin, ECOG: Eastern Cooperative Oncology Group, eGFR: estimated glomerular filtration rate, FAN: Fib-4 index, ALBI score and NLR, HR: hazard ratio, CI: confidence interval.

We examined the usefulness of the FAN score stratified by ECOG PS score. In 80 patients with ECOG PS of 1, the prognosis of high risk group of FAN score was significantly poorer about PFS (p = 0.018) and CSS (p < 0.001) (Supplemental Fig. 2c,d). Also, in 27 patients with ECOG PS of 2, the prognosis of high risk group of FAN score was significantly poorer about PFS (p = 0.049) (Supplemental Fig. 2e).

### Validation of the clinical importance of the FAN score in the independent cohort

In the validation cohort, the median PFS durations of the low-risk and high-risk groups were 4.37 months (95% CI 2.94–5.80) and 1.87 months (95% CI 0.46–3.29), respectively (p = 0.014) (Fig. 3c). Moreover, the median CSS durations of the low-risk and high-risk groups were 11.6 months (95% CI 8.08–15.2) and 3.62 months (95% CI 2.21–5.03), respectively (p = 0.010) (Fig. 3d).

Regarding PFS, poor ECOG PS, the presence of liver metastasis, and high-risk group based on the FAN score were associated with poor prognosis in univariate analysis (Table 5). In multivariate analysis, poor ECOG PS (HR: 2.80; 95% CI 1.33–5.86, p = 0.006), the presence of liver metastasis (HR: 2.14; 95% CI 1.01–4.52, p = 0.047), high-risk group based on the FAN score (HR: 1.29, 95% CI 1.02–1.62, p = 0.034) were significant prognostic factors for predicting an inferior PFS (Table 5).

**Table 5:** Univariate and multivariate analysis of predictive factors for PFS in validation cohort (n = 103).

	Univariate				Multivariate			
	HR	95% CI		p	HR	95%CI		p
		Lower	Higher			Lower	Higher	
**Age**								
< 73 vs. ≥ 73	1.43	0.91	2.23	0.119	1.26	0.79	2.00	0.329
**Sex**								
Male vs. female	0.95	0.58	1.55	0.832	0.89	0.53	1.47	0.637
**Primary site**								
Upper urinary tract involvement vs. others	0.73	0.47	1.14	0.164				
**Surgical removal of primary site**								
No vs. yes	0.96	0.56	1.65	0.885				
**Previous chemotherapy regimen**								
Cisplatin vs. non-cisplatin	0.84	0.54	1.31	0.448				
**Time from last chemotherapy (days)**								
≥ 90 vs. < 90	1.14	0.72	1.82	0.577				
**Hb (g/dl)**								
≥ 10.0 vs. < 10.0	1.46	0.94	2.29	0.096				
**ECOG performance status**								
0 vs. 1	1.35	0.73	2.50	0.346	1.20	0.62	2.35	0.474
0 vs. ≥ 2	3.63	1.80	7.29	< 0.001	2.80	1.33	5.86	0.006
**Metastatic sites**								
Lymphnode vs. other organs	1.71	0.91	3.23	0.096	1.55	0.79	3.04	0.625
Lymphnode vs. liver metastasis	2.84	1.38	5.83	0.004	2.14	1.01	4.52	0.047
**eGFR (ml/min/1.73 m**^**2**^**)**								
≥ 60 vs. 30.0 ≤ , < 60.0	0.97	0.57	1.67	0.923				
≥ 60 vs. < 30	0.74	0.35	1.56	0.735				
**FAN score**								
Low risk group vs. high risk group	1.33	1.06	1.67	0.015	1.29	1.02	1.62	0.034

Hb: hemoglobin, ECOG: Eastern Cooperative Oncology Group, eGFR: estimated glomerular filtration rate, FAN: Fib-4 index, ALBI score and NLR, HR: hazard ratio, CI: confidence interval.

Regarding CSS, poor ECOG PS, the presence of liver metastasis, and high-risk group based on the FAN score were associated with poor prognosis in univariate analysis (Table 5). In multivariate analysis, poor ECOG PS (HR: 3.87; 95% CI 1.46–10.27, p = 0.007), the presence of liver metastasis (HR: 3.60; 95% CI 1.37–9.51, p = 0.010), and high-risk group based on the FAN score (HR: 1.48, 95% CI 1.19–1.85, p = 0.001) were significant prognostic factors for predicting an inferior PFS (Table 6).

**Table 6:** Univariate and multivariate analysis of predictive factors for CSS in validation cohort (n = 103).

	Univariate				Multivariate			
	HR	95% CI		p	HR	95%CI		p
		Lower	Higher			Lower	Higher	
**Age**								
< 73 vs. ≥ 73	1.41	0.84	2.38	0.195	1.32	0.77	2.27	0.308
**Sex**								
Male vs. female	0.83	0.46	1.47	0.516	0.85	0.46	1.56	0.603
**Primary site**								
Upper urinary tract involvement vs. others	0.87	0.52	1.47	0.598				
**Surgical removal of primary site**								
No vs. yes	1.36	0.68	2.70	0.382				
**Previous chemotherapy regimen**								
Cisplatin vs. non-cisplatin	0.79	0.47	1.34	0.384				
**Time from last chemotherapy (days)**								
≥ 90 vs. < 90	1.18	0.68	2.04	0.557				
**Hb (g/dl)**								
≥ 10.0 vs. < 10.0	1.93	1.15	3.25	0.013	1.50	0.86	2.63	0.152
**ECOG performance status**								
0 vs. 1	1.86	0.78	4.45	0.163	1.59	0.64	3.91	0.316
0 vs. ≥ 2	5.66	2.25	14.26	< 0.001	3.87	1.46	10.27	0.007
**Metastatic sites**								
Lymphnode vs. other organs	2.39	1.00	5.68	0.049	1.86	0.77	4.54	0.167
Lymphnode vs. liver metastasis	5.11	2.00	13.03	0.001	3.60	1.37	9.51	0.010
**eGFR (ml/min/1.73 m**^**2**^**)**								
≥ 60 vs. 30.0 ≤ , < 60.0	0.69	0.38	1.26	0.227				
≥ 60 vs. < 30	0.74	0.33	1.68	0.741				
**FAN score**								
Low risk group vs. high risk group	1.41	1.08	1.84	0.011	1.48	1.19	1.85	0.001

Hb: hemoglobin, ECOG: Eastern Cooperative Oncology Group, eGFR: estimated glomerular filtration rate, FAN: Fib-4 index, ALBI score and NLR, HR: hazard ratio, CI: confidence interval.

About clinical therapeutic response, ORR was 28.8% and 33.3% in low-risk and high-risk group based on the FAN score, respectively. Also, DCR was 53.5% and 40.0% in low-risk and high-risk group based on the FAN score, respectively and there were no significant differences about ORR (p = 0.644) and DCR (p = 0.279) between low-risk and high-risk group based on FAN score (Supplemental Table 3).

We examined the usefulness of the FAN score stratified by ECOG PS score. In 23 patients with ECOG PS of 2, the prognosis of high risk group of FAN score was significantly poorer about PFS (p = 0.018) and CSS (p < 0.001) (Supplemental Fig. 3e,f).

Although ECOG PS is well established prognostic factor, the FAN score could predict prognosis more accurately in some patient groups.

## Discussion

Various prognostic factors of patients with mUC have been reported. They were mainly blood test results related to systemic inflammation^8–11^. Additionally, clinical items reflecting cancer-related cachexia have been mainly reported. Further, the presence of liver metastasis has been reported as a poor prognostic factor for patients with mUC treated with either chemotherapy^19^ or immunotherapy^8–12^. Although these previous studies used the same blood test results, the cut-off values of each item were different according to each cohort and their versatility was low. The FAN score established herein was a significant prognostic factor of CSS and PFS in two independent cohorts, and it was based on only blood test results using the previous established cut-off points.

Among the elements comprising our established FAN score, the Fib-4 index was established as a scoring system for predicting liver fibrosis in patients with viral hepatitis and non-alcoholic fatty liver^15^. A score of ≥ 3.5 is used as a non-invasive indicator of liver cirrhosis^17^. Median Fib-4 in the discovery cohort in our study was 1.673, and it was significantly lower than cut-off value compared to the other several reports. We adopted to use 3.5 as a cut-off point because it was the value of 90th percentile in the discovery cohort and it could stratify the prognosis. The ALBI score is as useful as the Child–Pugh grade as a liver reserve scoring system for patients with hepatocellular carcinoma (HCC)^16^. The score is based on the serum albumin and total bilirubin levels, and the cut-off of − 2.6 is widely accepted. Since the median value of ALBI was − 2.56 in the discovery cohort of this study, we adopted − 2.6 as cut-off value, which was established for HCC. The NLR has been often reported as a prognostic factor for various types of cancer, but its cut-off value has not been established. For UC, cut-off values of 3.0^10,11^ and 5.0^8^ have been reported, but in our study, we used 5.0 because this value was used in a previous study with a large number of cases.

In our study, we were able to show that a combination of the aforementioned three items is the most suitable scoring system for CSS and PFS in mUC, even when compared with other items such as the CRP level, which has already been reported^11^. The fact that the FAN score could be validated in two independent cohorts supports the versatility of this scoring system.

The Fib-4 index and ALBI score used in the present study are not only scoring systems for hepatic function^15,17^, but they have also recently been reported as prognostic factors for cancer immunotherapy for hepatocellular carcinoma^20–22^, cardiac disease^23,24^ and coronavirus disease^25,26^. Additionally, they are presumed to be scores that reflect general conditions related to cachexia. Cancer-related cachexia is thought to be caused by changes in the systemic metabolic environment due to elevated inflammatory cytokines such as IL-6 and TNF^27,28^ in adipose tissue^29,30^, tumour cells^31^, and hepatocytes^32^; changes in protein synthesis and degradation in skeletal muscle; and insulin resistance^33^, which together lead to weight loss, anorexia, decreased systemic function, and increased adiposity^34^.

We could not find a significant correlation between the presence of liver metastasis and the Fib-4 index or ALBI score. The main reason for this may be the low diagnostic accuracy of simple computed tomography used in daily clinical practice for evaluating liver metastasis. In our cohort, it significantly correlated both ALBI score and NLR with cachexia related blood tests such as anaemia (p < 0.001) and CRP level (p < 0.001). Whereas, the Fib-4 index was significantly correlated with sex (p = 0.013) and age (p = 0.028) and it was not significantly correlated with cachexia related blood tests. There were no significant correlations between the Fib-4 index and ALBI score or NLR and these results suggest FAN score can include 3 scoring systems reflecting different liver dysfunctions. In the future, it will be important to elucidate the mechanism of changes in the Fib-4 index and ALBI score related to liver function decline caused by aging and cancer-associated cachexia, and to develop novel treatments to improve the therapeutic effect of ICI therapy.

In the present study, there was almost no difference in the CSS and PFS between the two cohorts. These results were similar to the outcomes of a phase III clinical trial^2^ and other large cohort studies^10^. Herein, the median CSS durations of the patients classified in the high-risk group based on the FAN score were 3.9 months and 3.6 months, which were significantly worse than those of the patients classified in the low-risk group. Of course, about 10% of patients in the high-risk group also had long-term survival, so we cannot conclude that physicians should not treat patients with a high FAN score. However, it is necessary to develop biomarkers for each patient group based on this score to provide more appropriate medication to patients in the future.

In the current study, we did not examine the association between the incidence of adverse drug reactions and the FAN score. At the very least, there was no correlation between the FAN score and cases of drug discontinuation due to side effects.

There are several limitations to our study. These include the fact that this was a retrospective analysis, the number of cases was small, and the blood collection items were limited to those used in daily clinical practice.

## Conclusions

In conclusion, we created the FAN score consisting of the Fib-4 index, ALBI score, and NLR based on blood test results. Using two independent cohorts, we found that the FAN score was a significant prognostic predictor in patients with mUC treated with pembrolizumab.

## Supplementary Information

Supplementary Information.

## Data Availability

Detailed data would remain confidential and was not allowed to be shared in our institutional review board.
