# Supplementary material for: FAN score comprising fibrosis-4 index, albumin–bilirubin score and neutrophil–lymphocyte ratio is a prognostic marker of urothelial carcinoma patients treated with pembrolizumab
Source: Sci Rep. 2021 Oct 27;11:21199. doi: 10.1038/s41598-021-00509-x (PMC8551158; doi:10.1038/s41598-021-00509-x)

**Manuscript title:** FAN score comprising fibrosis-4 index and albumin-bilirubin score is a prognostic marker of urothelial carcinoma patients treated with pembrolizumab

Atsunari Kawashima^a*^, Yoshiyuki Yamamoto^b^, Mototaka Sato^c^, Wataru Nakata^d^, Yoichi Kakuta^e^, Yu Ishizuya^f^, Yuichiro Yamaguchi^g^, Akinaru Yamamoto^h^, Takahiro Yoshida^i^, Hitoshi Takayama^j^, Tsuyoshi Takada^k^, Hitoshi Inoue^l^, Yohei Okuda^m^, Taigo Kato^a^, Koji Hatano^a^, Motohide Uemura^a^, Norio Nonomura^a^, and Ryoichi Imamura^a^

^a^Department of Urology, Osaka University Graduate School of Medicine, Osaka, Japan

^b^Department of Urology, Osaka International Cancer Institute

^c^ Department of Urology, Toyonaka Municipal Hospital

^d^ Department of Urology, Osaka Rosai Hospital

^e^ Department of Urology, Osaka General Medical Center

^f^ Department of Urology, Higashiosaka City Medical Center

^g^Department of Urology, JCHO Osaka Hospital

^h^Department of Urology, Osaka Police Hospital

^i^Department of Urology, Hyogo Prefectural Nishinomiya Hospital

^j^Department of Urology, Sakai City Medical Center

^k^Department of Urology, Minoh City Hospital

^l^Department of Urology, Ikeda City Hospital

^m^Department of Urology, Sumitomo Hospital

Correspondence to Atsunari Kawashima, MD, PhD

Department of Urology, Osaka University

2-2 Yamadaoka, Suita City, Osaka 565-0871, Japan

TEL: +81-6-6879-3531; FAX: +81-6-6879-3539

E-mail: [kawashima@uro.med.osaka-u.ac.jp](mailto:kawashima@uro.med.osaka-u.ac.jp)

**Supplemental Figure Legends**

Fig. 1: Research flow on data collection and analysis in this study

Fig. 2: Probability estimates of the prognosis of study patients with metastatic urothelial carcinoma who were treated with pembrolizumab and stratified by the FAN score in the discovery cohort. Probability estimates of progression-free survival (PFS) (a) and cancer-specific survival (CSS) (b) for the 58 patients with ECOG PS 0. Probability estimates of PFS (c) and CSS (d) for the 80 patients with ECOG PS 0. Probability estimates of PFS (c) and CSS (d) for the 27 patients with ECOG PS 2.

Fig. 3: Probability estimates of the prognosis of study patients with metastatic urothelial carcinoma who were treated with pembrolizumab and stratified by the FAN score in the verification cohort. Probability estimates of progression-free survival (PFS) (a) and cancer-specific survival (CSS) (b) for the 22 patients with ECOG PS 0. Probability estimates of PFS (c) and CSS (d) for the 58 patients with ECOG PS 0. Probability estimates of PFS (c) and CSS (d) for the 23 patients with ECOG PS 2.

**Supplemental Table 1 – Clinical values of blood tests used to establish prognostic model and steps of**

**Cox regression stepwise backward analysis.**

|  | Clinical values | | HR | Lower  95% CI | Higher  95% CI | p-value |
| --- | --- | --- | --- | --- | --- | --- |
| STEP  1 | Hb | (≥10.0 g/dl vs. <10.0 g/dl) | 1.242 | 0.784 | 1.968 | 0.356 |
|  | Plt | (>32.0*10^4^ /μl vs. ≤32.0*10^4^ /μl) | 1.071 | 0.567 | 2.024 | 0.832 |
|  | AST | (>40 U/L vs. ≤40 U/L) | 1.989 | 0.709 | 5.578 | 0.192 |
|  | ALT | (>40 U/L vs. ≤40 U/L) | 0.792 | 0.271 | 2.318 | 0.671 |
|  | Serum Sodium | (≥138 mEq/L vs. <138 mEq/L) | 0.836 | 0.546 | 1.279 | 0.408 |
|  | eGFR | (≥30 ml/min/1.73m^2^ vs. <30 ml/min/1.73m^2^) | 1.174 | 0.896 | 1.538 | 0.244 |
|  | Albumin | (≥3.5 mg/dl vs. <3.5 mg/dl) | 0.945 | 0.477 | 1.874 | 0.871 |
|  | CRP | (≥1.0 mg/dl vs. <1.0 mg/dl) | 1.369 | 0.862 | 2.174 | 0.183 |
|  | NLR | (≥5.0 vs. <5.0) | 1.508 | 0.874 | 2.602 | 0.140 |
|  | MLR | (≥0.43 vs. <0.43) | 0.702 | 0.424 | 1.163 | 0.170 |
|  | PLR | (≥1.23 vs. <1.23) | 1.475 | 0.829 | 2.623 | 0.186 |
|  | Fib-4 index | (≥3.5 vs. <3.5) | 1.880 | 0.878 | 4.022 | 0.104 |
|  | ALBI score | (>-2.6 vs. ≤-2.6) | 1.447 | 0.871 | 2.402 | 0.153 |
|  |  |  |  |  |  |  |
| STEP 6 | AST | (>40 U/L vs. ≤40 U/L) | 1.839 | 0.754 | 4.488 | 0.181 |
|  | CRP | (≥1.0 mg/dl vs. <1.0 mg/dl) | 1.347 | 0.858 | 2.117 | 0.196 |
|  | NLR | (≥5.0 vs. <5.0) | 1.619 | 0.969 | 2.704 | 0.066 |
|  | MLR | (≥0.43 vs. <0.43) | 0.737 | 0.447 | 1.215 | 0.231 |
|  | PLR | (≥1.23 vs. <1.23) | 1.522 | 0.888 | 2.607 | 0.126 |
|  | Fib-4 index | (≥3.5 vs. <3.5) | 2.036 | 0.984 | 4.213 | 0.055 |
|  | ALBI score | (>-2.6 vs. ≤-2.6) | 1.614 | 1.010 | 2.580 | 0.046 |
| STEP 7 | AST | (>40 U/L vs. ≤40 U/L) | 1.822 | 0.740 | 4.488 | 0.192 |
|  | CRP | (≥1.0 mg/dl vs. <1.0 mg/dl) | 1.317 | 0.839 | 2.067 | 0.231 |
|  | NLR | (≥5.0 vs. <5.0) | 1.504 | 0.915 | 2.472 | 0.108 |
|  | PLR | (≥1.23 vs. <1.23) | 1.348 | 0.813 | 2.234 | 0.248 |
|  | Fib-4 index | (≥3.5 vs. <3.5) | 1.859 | 0.904 | 3.824 | 0.092 |
|  | ALBI score | (>-2.6 vs. ≤-2.6) | 1.543 | 0.969 | 2.455 | 0.067 |
| STEP 8 | AST | (>40 U/L vs. ≤40 U/L) | 1.796 | 0.735 | 1.389 | 0.199 |
|  | CRP | (≥1.0 mg/dl vs. <1.0 mg/dl) | 1.356 | 0.867 | 2.121 | 0.182 |
|  | NLR | (≥5.0 vs. <5.0) | 1.753 | 1.137 | 2.704 | 0.011 |
|  | Fib-4 index | (≥3.5 vs. <3.5) | 1.646 | 0.831 | 3.260 | 0.153 |
|  | ALBI score | (>-2.6 vs. ≤-2.6) | 1.602 | 1.011 | 2.536 | 0.045 |
| STEP 9 | CRP | (≥1.0 mg/dl vs. <1.0 mg/dl) | 1.363 | 0.872 | 2.133 | 0.175 |
|  | NLR | (≥5.0 vs. <5.0) | 1.714 | 1.115 | 2.636 | 0.014 |
|  | Fib-4 index | (≥3.5 vs. <3.5) | 2.022 | 1.136 | 3.599 | 0.017 |
|  | ALBI score | (>-2.6 vs. ≤-2.6) | 1.686 | 1.073 | 2.650 | 0.024 |
| STEP 10 | NLR | (≥5.0 vs. <5.0) | 1.842 | 1.216 | 2.791 | 0.004 |
|  | Fib-4 index | (≥3.5 vs. <3.5) | 2.127 | 1.202 | 3.764 | 0.010 |
|  | ALBI score | (>-2.6 vs. ≤-2.6) | 1.911 | 1.266 | 2.884 | 0.002 |

**Supplemental Table 2 - Clinical responses to pembrolizumab therapy in Discovery (n=165) and Validation (n=103) cohort.**

| Clinical response | Discovery Cohort | Validation Cohort |
| --- | --- | --- |
| CR | 14 (8.5%) | 4 (3.9%) |
| PR | 22 (13.3%) | 27 (26.2%) |
| SD | 28 (17.0%) | 20 (19.4%) |
| PD | 101 (61.2%) | 52 (50.5%) |
| ORR (CR+PR) | 36 (21.8%) | 31 (30.1%) |
| DCR (CR+PR+SD) | 64 (38.8%) | 51 (49.5%) |

CR: complete response; PR: partial response; SD: stable disease; PD: progressive disease; ORR: objective response rate; DCR: disease control rate.

**Supplemental Table 3 - Clinical responses to pembrolizumab therapy in Discovery (n=165) and Validation (n=103) cohort stratified by FAN score**

|  | Discovery Cohort | | | Validation Cohort | | | | |
| --- | --- | --- | --- | --- | --- | --- | --- | --- |
| Clinical response | FAN score  Low  (n=116) | FAN score High  (n=49) | p | FAN score  Low  (n=73) | FAN score  High  (n=30) | p | |  |
| CR | 13 (11.2%) | 1 ( 2.0%) |  | 3 ( 4.1%) | 1 ( 3.3%) | |  | |
| PR | 17 (14.7%) | 5 (10.2%) |  | 18 (24.7%) | 9 (30.0%) | |  | |
| SD | 26 (22.4%) | 2 ( 4.1%) |  | 18 (24.7%) | 2 ( 6.7%) | |  | |
| PD | 60 (51.7%) | 41 (83.7%) |  | 34 (46.5%) | 18 (60.0%) | |  | |
| ORR (CR+PR) | 30 (25.9%) | 6 (12.2%) | 0.064 | 21 (28.8%) | 10 (33.3%) | | 0.644 | |
| DCR (CR+PR+SD) | 56 (48.3%) | 8 (16.3%) | < 0.001 | 39 (53.5%) | 12 (40.0%) | | 0.279 | |

CR: complete response; PR: partial response; SD: stable disease; PD: progressive disease; ORR: objective response rate; DCR: disease control rate.


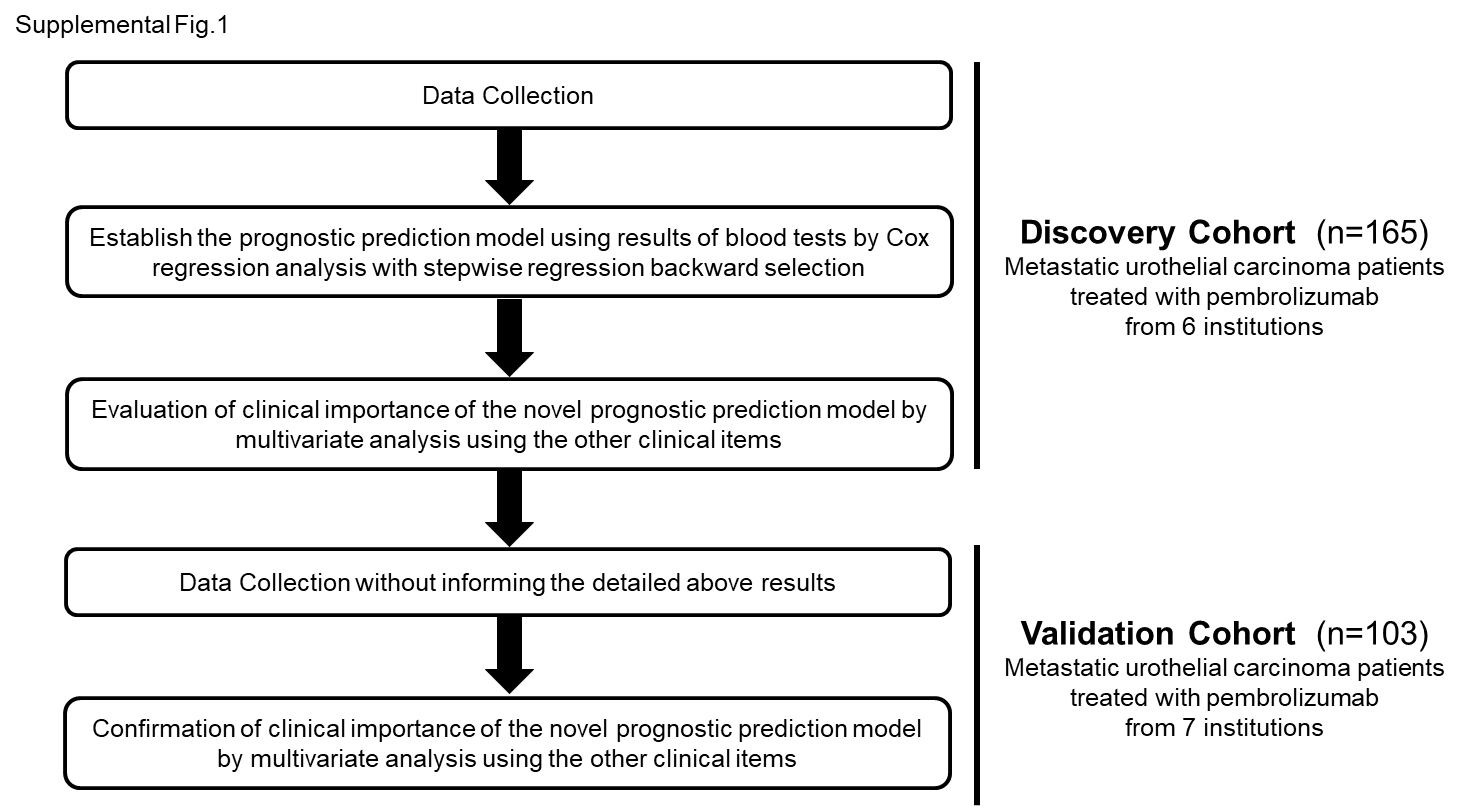


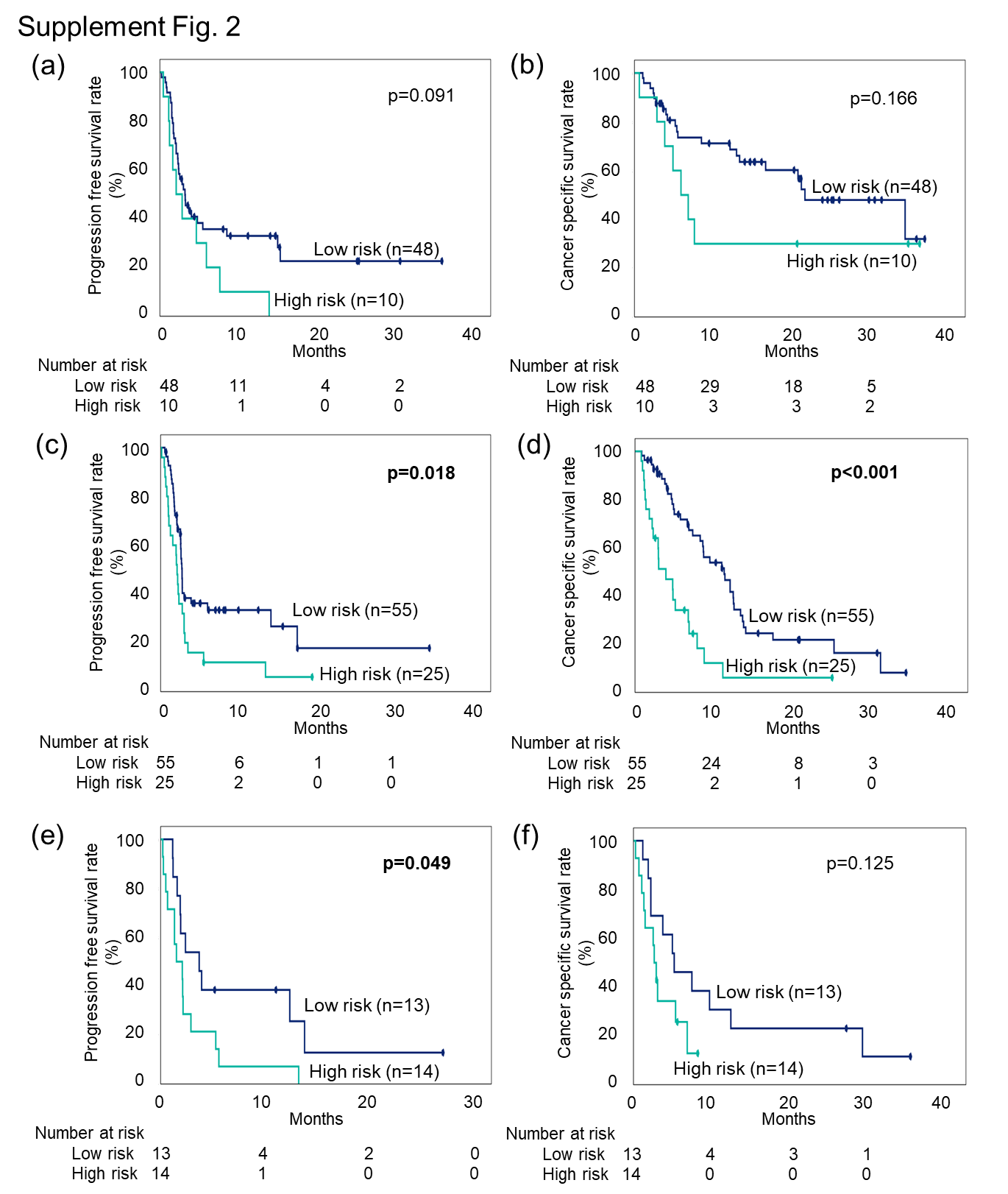


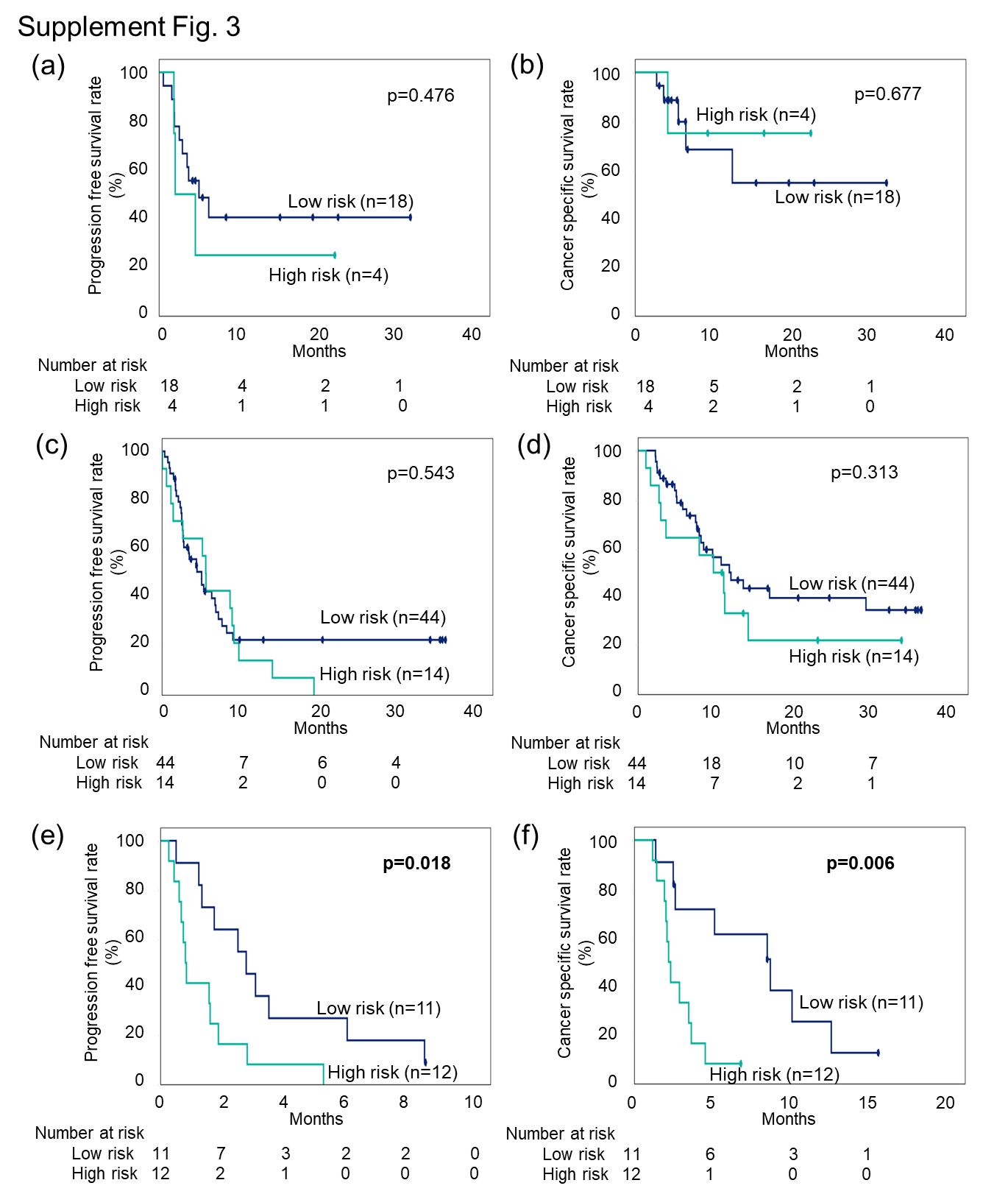

Supplement: Supplementary file 1 — Supplementary Information. [file 41598_2021_509_MOESM1_ESM.docx]
